# Predictive Biomarkers for Coronary Collateral Circulation Development After Myocardial Infarction

**DOI:** 10.3390/ijms27083671

**Published:** 2026-04-20

**Authors:** Andrei Constantinescu, Miruna Mihaela Micheu, Elisa Anamaria Liehn, Alexandru Scafa Udrişte

**Affiliations:** 1 Center of Innovation and e-Health, University of Medicine and Pharmacy “Carol Davila”, 20, Pitar Moș, 030167 Bucharest, Romania; andrei.constantinescu@umfcd.ro (A.C.); alexandru.scafa@umfcd.ro (A.S.U.); 2 Department of Cardiology, University of Medicine and Pharmacy “Carol Davila”, 8, Eroii Sanitari, 050474 Bucharest, Romania; 3 Department of Cardiology, Clinical Emergency Hospital of Bucharest, 8, Calea Floreasca, 014461 Bucharest, Romania; 4 Centre of Excellence PREPARE, Clinical Emergency Hospital of Bucharest, 8, Calea Floreasca, 014461 Bucharest, Romania; 5 National Heart Center Singapore, 5, Hospital Dr, Singapore 169609, Singapore; elisa.liehn@outlook.de

**Keywords:** collateral coronary circulation, cardiovascular biomarkers, myocardial infarction, arteriogenesis, microRNAs

## Abstract

Myocardial infarction remains a leading cause of mortality worldwide as the most severe clinical presentation of coronary artery disease, with an increasing trend in young adults. In the early phase of myocardial infarction, the mean blood pressure regulates the pressure distal to the occluded artery in the presence of well-developed collateral coronary circulation. Hypotensive medication administered after the myocardial infarction could compromise collateral recruitment and exacerbate myocardial ischemia. Collateral coronary circulation develops through angiogenic processes as a network of small blood vessels. After the myocardial infarction, the collateral arteries open and begin a process of arteriogenesis in order to mature into functional arteries. Although there are several well-known biochemical and molecular biomarkers for both myocardial infarction and angiogenesis, we need to associate these with arteriogenesis biomarkers in order to be able to fully determine the level of collateral coronary circulation development after myocardial infarction. In this review, we summarize some of the most important biomarkers that could provide insight into the collateral coronary arteriogenesis process. Our aim is to identify specific biomarkers that can be identified in the early processes of arteriogenesis after the myocardial event in order to quickly determine the best therapeutic strategy.

## 1. Introduction

Cardiovascular diseases (CVDs) continue to be the main cause of morbidity and mortality around the world, with ischemic heart disease being the most prevalent cause of mortality [1]. Myocardial infarction (MI) is a high-prevalence life-threatening coronary event and the most severe clinical presentation of coronary artery disease (CAD) [2], with many contributing risk factors, such as health behaviors (smoking, physical inactivity, nutrition, sleep and obesity) and health factors (cholesterol, blood pressure, glucose control and metabolic syndrome) [3]. In the last decade, acute myocardial infarction (AMI) has also shown an increasing trend in young adults [4]. In the early phase of MI, the mean blood pressure regulates the pressure distal to the occluded artery in the presence of functioning coronary collateral circulation (CCC) [5]. Therefore, it is important to determine if such CCC is well developed or not when administering hypotensive therapies during an AMI, as rapid reductions in systemic blood pressure could compromise collateral recruitment and exacerbate myocardial ischemia [6].

**Figure 1:**
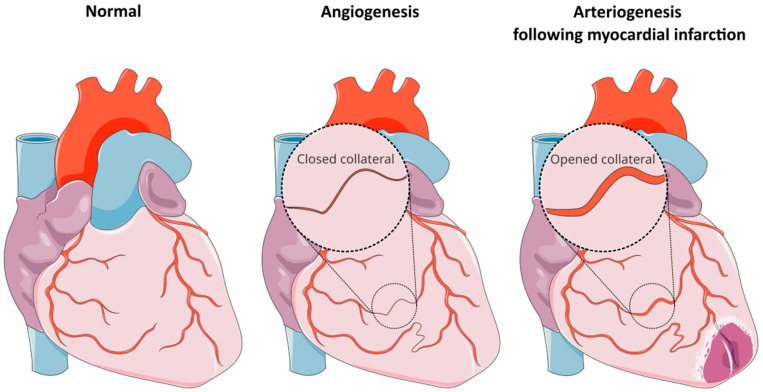
Processes of angiogenesis and arteriogenesis. Adapted from Servier Medical Art (https://smart.servier.com), licensed under CC BY 4.0 (https://creativecommons.org/licenses/by/4.0/).

Angiogenesis represents the formation of new capillary blood vessels, while arteriogenesis represents an increase in the diameter and maturation of arterial vessels. CCC is created through angiogenesis as a primitive network of blood vessels that have the ability to augment in size and function following a myocardial infarction, in order to allow blood flow between the two arteries (Figure 1).

**Figure 2:**
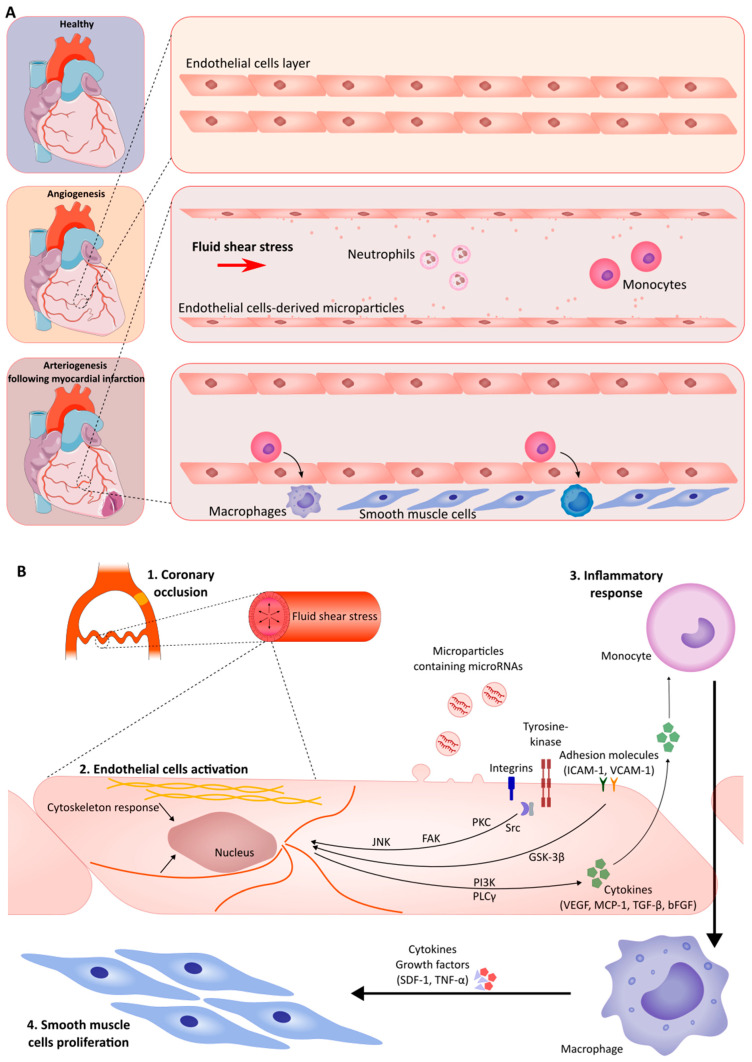
(**A**) Overview of coronary collateral circulation activation following coronary occlusion. Adapted from Servier Medical Art (https://smart.servier.com), licensed under CC BY 4.0 (https://creativecommons.org/licenses/by/4.0/). (**B**) Cells and molecular events in arteriogenesis.

In the process of arteriogenesis, as a primary mechanism of CCC development, the collateral arteries open and begin developing and maturing initially as a result of increased fluid shear stress (FSS) (Figure 2A). Afterwards, several processes are initiated within the collateral blood vessels, such as endothelial cell (EC) activation, inflammatory cell accumulation, and the activation of a multitude of chemokines and growth factors, which promote EC and smooth muscle cell (SMC) proliferation (Figure 2B).

Once the collateral arteries have increased their size and caliber, there is negative feedback loop signaling, resulting in a decrease in molecular modifications and a reduction in the initial hypoxic microenvironment required to initiate angiogenesis and after MI for CCC development through arteriogenesis [7]. Both ECs and SMCs within the collateral arteries are specialized, making them distinct from those in other blood vessels; they maintain collateral integrity during exposure to factors such as shear stress and low blood oxygen. For example, collateral ECs are aligned with the vessel axis, unlike the cobble-stoned morphology of ECs exposed to low shear stress. Moreover, collateral ECs present primary cilia, which sense shear stress [8]. The mechanisms of collateral circulation development are complex and depend on several factors besides mechanical stress. Chemical factors play a central role in a multitude of processes. ECs initially secrete monocyte chemoattractant protein-1 (MCP-1), initiating correct monocyte migration and subsequent attachment via intracellular adhesion molecule-1 (ICAM-1) and vascular cell adhesion molecule-1 (VCAM-1). This is followed by the differentiation of monocytes into macrophages—specifically, pro-angiogenic M2 macrophages, which promote SMC growth and differentiation. Vascular endothelial growth factor (VEGF), a major driver of both angiogenesis and arteriogenesis, is involved in facilitating the survival, migration, and proliferation of cells by activating several signaling pathways. In addition to VEGF, basic fibroblast growth factor (bFGF) and platelet-derived growth factor (PDGF) are implicated in arteriogenesis during CCC development due to their mitogenic effects, promoting cell migration and differentiation. Moreover, nitric oxide (NO), produced by the activity of endothelial nitric oxide synthase (eNOS), increases vasculature permeability, promoting macrophage invasion. Another notable factor is early growth response 1 (Egr-1), a transcription factor that is upregulated during arteriogenesis, controlling the expression of PDGF and transforming growth factor beta (TGF-β), which is then indirectly involved in upregulating the expression of other factors, such as VEGF and metalloproteinases (MMPs) [9]. Clinical data have shown that shear stress can induce arteriogenesis via external counterpulsation therapy (ECP) in patients with stable CAD. This was based on increased levels of TGF-β and platelet-derived microparticles (PMPs) [10].

## 2. Methods

### 2.1. Literature Search

The literature search was performed on the PubMed database to retrieve potentially eligible studies. The search timeframe was from the establishment of the database to 2026. The keywords used were “coronary collateral circulation”, “arteriogenesis”, “angiogenesis”, “myocardial infarction”, “biomarkers”, and “microRNA”.

### 2.2. Eligibility Criteria

Articles meeting the following criteria were included: (1) studies including patients with CAD, CTO, ASC, or AMI; (2) studied related to microRNAs and serological biomarkers such as cytokines, chemokines, and growth factors; (3) studies related to coronary collateral development following myocardial infarction. Studies excluded from this review were (1) case studies and conference abstracts; (2) studies published in languages other than English.

### 2.3. Selection Strategy

As a final selection strategy, articles were selected based on the latest data available that matched the search criteria and inclusion/exclusion eligibility criteria, and we focused on biomarkers that are involved in functional modifications related to the arteriogenic process following myocardial infarction.

## 3. Cellular and Molecular Response

In this section, we review the main cellular and molecular events that occur during the arteriogenesis process following myocardial infarction during coronary collateral vascularization based on in vitro and in vivo experimental data.

### 3.1. Shear Stress

Endothelial cells respond to mechanical forces by activating cell signaling pathways. There is recent evidence that an increase in metabolism occurs in order to increase eNOS activity, reinforce the actin cytoskeleton and cadherin adhesion complex, and enhance glucose uptake as a response to the shear stress of the mechanical force applied. These processes occur via the VE-cadherin-stimulated AMP-activated protein kinase (AMPK), a master regulator of energy homeostasis [11]. Endothelial cells in the descending aorta show a response to high laminar shear stress via the expression of transient receptor potential vanilloid (TRPV4) ion channels on the plasma membrane, which mediate Ca^2+^ entry by physically interacting with caveolin-1 clusters in localized microdomains. Signaling via these channels reduced the inflammatory response and promoted cell resilience [12]. Moreover, a different study identified three hub genes as potential mechanosensitive genes in human blood vessels: *ATF3*, *HSPA6*, and *DUSP1*. ATF3 is a transcription factor belonging to the activated protein 1 (AP-1) family, with an important role in the reduction of inflammatory genes, and it is found in high-laminar-flow blood vessels. HSPA6 (Hsp70B) is a member of the HSPA (Hsp70) multigene family, correlated with endothelial dysfunction and atherosclerotic vascular lesions. Dual-specificity protein phosphatase 1 (DUSP1) is a nuclear dual-specific phosphatase involved in the immunomodulation of senescent ECs [13]. Moreover, FSS, as a key initiating factor in arteriogenesis, increases the expression of placental growth factor (PLGF) in human coronary artery ECs in vitro via the action of heme oxygenase 1 (HO-1) [14], an enzyme under the control of the Nrf2 master transcriptional regulator, which can have both pro- and anti-inflammatory effects [15]. Nrf2 activity is regulated via AMPK/GSK-3β [16], an important signaling axis involved in several processes that occur under FSS.

### 3.2. Microparticles

Microvesicles, or microparticles (MPs), range in size from 100 nm to 1000 nm. They are formed through the outward budding of the cells’ plasma membrane via a calcium-dependent mechanism that induces actin cytoskeleton modifications [17]. Produced by all cell types in the body, MPs serve as carriers for bioactive cargo, including cytokines, enzymes, cell receptors, and microRNAs. MPs have an active role in paracrine signaling and can also be used as markers for various CVDs [18]. For example, platelet-derived microparticles have been shown to upregulate the expression of E-selectin, VCAM-1, ICAM-1, and PECAM-1 in endothelial cells in vitro [19]. Moreover, EC-derived MPs (EMPs) from patients with vascular intimal hyperplasia promoted ERK and p38 phosphorylation and the increased production of ICAM-1 and VCAM-1 [20].

### 3.3. Endothelial Cell Cytoskeleton

Under FSS, the EC morphology changes, initiating rearrangements of its cytoskeleton structure and nuclear shape to adapt to mechanical stress and maintain vascular homeostasis. P300 is a lysine acetyltransferase that acts as a key regulator of the nuclear acetylome. Alongside several important vascular gene targets related to high laminar stress, such as eNOS, P300 is also involved in the expression regulation of other mechanotransduction genes, like *KLF4* and *YAP*, and the perinuclear actin caps, pointing towards an important role in modulating the EC cytoskeleton response to hemodynamic shear stress [21]. Angiomotin-like 2 (*AmotL2*), a target gene of YAP, is a junction mechanotransducer that connects cell–cell junctions to the nuclear membrane via the actin cytoskeleton. Mechanical stimuli activate a YAP-AmotL2 feedback loop of control over vascular homeostasis that helps to maintain the nuclear shape during FSS [22].

Alongside actin filament rearrangement, active microtubule remodeling is also required for EC elongation and alignment as a response to FSS. Microtubule acetylation expands from a Golgi-centric pattern and is regulated by HDAC6 and alpha-TAT1 [23]. This process is controlled by TGF-β-activated kinase 1 (TAK1) in response to TGF-β activation, which activates α-tubulin acetyltransferase 1 (α-TAT1) [24]. Another important component in microtubule rearrangement is the action of glycogen synthase kinase-3β (GSK-3β), a kinase that phosphorylates many microtubule plus-end tracking proteins, detaching them from the filaments, affecting microtubule growth and cross-linking with actin [25]. GSK-3β activity is inhibited by phosphorylation in response to blood flow [26], and it is negatively regulated by the PI3K/Akt signaling pathway [27].

**Figure 3:**
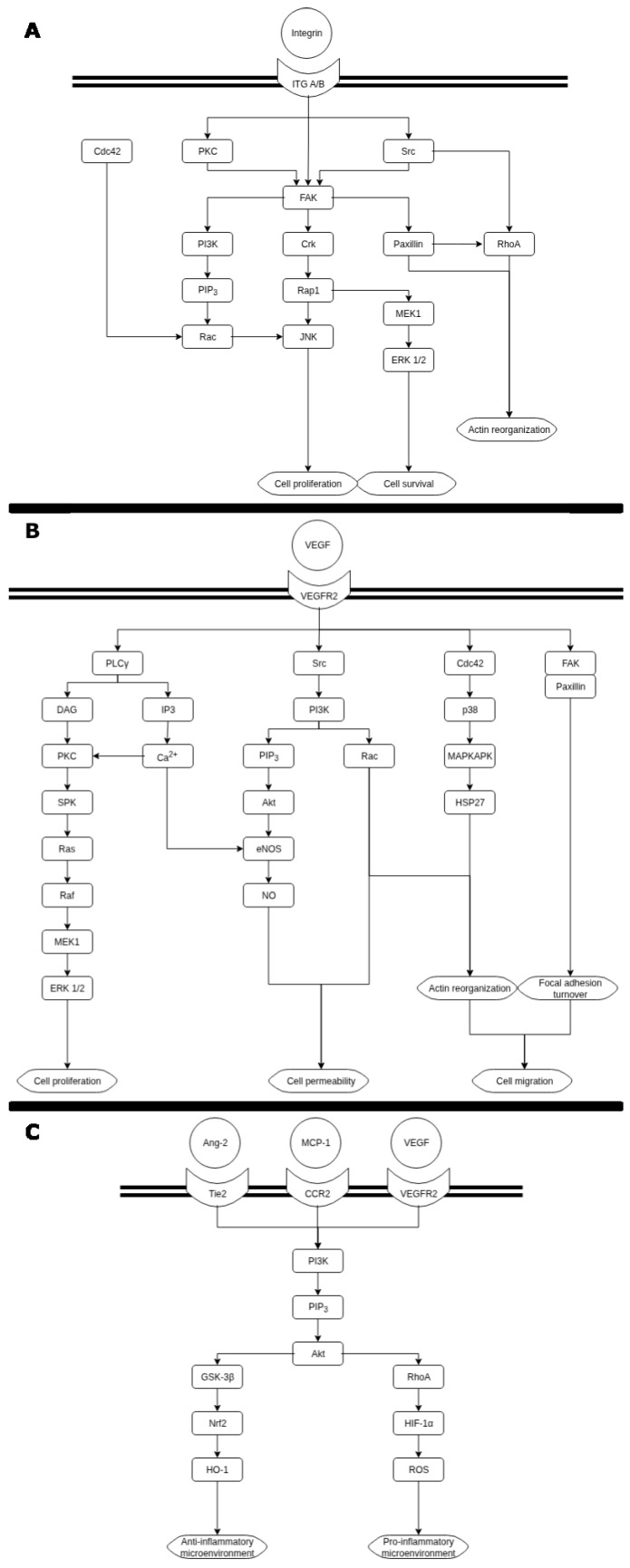
Main signaling pathways involved in arteriogenesis: (**A**) integrin–focal adhesion signaling; (**B**) NO production via VEGF signaling; (**C**) ROS production via HIF-1α signaling.

Following MI, the initial step in CCC arteriogenesis is the detection of elevated FSS by the mechanoreceptors within the endothelium. Shear stress signals are transmitted via integrin–focal adhesion kinase (FAK), with different end results. Integrins lead to the autophosphorylation of FAK and the phosphorylation of the FAK-associated protein Paxillin. FAK undergoes further Src-mediated hyperphosphorylation due to the recruitment and activation of Src to focal adhesions. In association with FAK/Src, Paxillin and Cas also become phosphorylated and in turn regulate cytoskeletal remodeling via Rap1, RhoA, CDC42, and Graf. Moreover, Rho and Cdc42 are linked with c-Jun N-terminal kinase (JNK) and subsequent c-Jun activation, leading to cell proliferation [28]. Exposure to acute shear stress activates ERK1/2 via the PKC-dependent MAPK cascade, which ultimately leads to cell survival. ERK activation is rapid and transient, leading to cell survival, while FAK/JNK activation is more sustained and leads to cell proliferation [29] (Figure 3).

The EphA2 kinase binds to FAK in resting cells and recruits PTPases upon stimulation with EphA1, a complex that negatively regulates FAK by dephosphorylation, thus reducing integrin-mediated cell adhesion [30]. EphB1 activation, in contrast, has been suggested to stimulate integrin-mediated cell adhesion [31].

### 3.4. Paracrine Signals

Following AMI, a series of pro- and anti-inflammatory cytokines are secreted in cascade, including pro-inflammatory cytokines such as INF-ϒ, TNF-α, IL-1, IL-6, and IL-18, but also angiogenic-promoting factors such as VEGF and PDGF. Moreover, key cytokines, such as IL-1, IL-6, IL-12, TNF-α, VEGF, MCP-1, and TGF-β, play a role in stimulating the migration and proliferation of coronary vascular ECs and vascular smooth muscle cells (VSMCs) and can promote or inhibit CCC development [32].

VEGF is a well-known major driver of angiogenesis and arteriogenesis. The development and maturation of CCC is no exception, where VEGF acts through interaction with one of its receptors, VEGF-R2, involved in activating signaling pathways such as FAK for focal adhesion migration, as well as DAG/PKC/ERK1/2 and IP3 for cell survival and proliferation or p38/MAPK for actin filament reorganization [33,34]. In the process of angiogenesis, the de novo growth of new capillaries is induced by hypoxia via HIF1-α activation and driven mainly by VEGF released by ischemic tissues and by inflammatory cells [35]. However, there is a multitude of genes related to inflammation, transcription, and neovascularization that are significantly upregulated during collateral growth [36]. It has been shown that CCC is impaired in patients with type II diabetes mellitus through multiple mechanisms involved in arteriogenesis and angiogenesis [37].

In addition to the activation of mechanoreceptors and pathways within the endothelium, there is also increased expression of molecules involved in monocyte attraction and adhesion, such as VCAM-1, MCP-1, and TGF-β. Once the immune cells adhere to the endothelium and enter the extracellular matrix (ECM), they differentiate into macrophages in order to create a favorable microenvironment for vessel growth and development, leading to the proliferation of VSMCs and contractile phenotype switching [7]. In vivo studies have also shown that AMPK in macrophages is an important regulator of arteriogenesis by directly phosphorylating IKKa and activating NF-kB, which functions as a transcription factor for TGF-β [38]. In turn, TFG-β is also a regulator of NF-kB via TAK1, leading to the AP-1 transcription factor via the JNK and p38 pathways [39,40].

Adropin, a peptide discovered in 2008, which was initially identified for its roles in energy balance, lipid metabolism, and glucose regulation, is also involved in cardiovascular functions, such as enhancing endothelial function, modulating lipid profiles, and reducing oxidative stress. Adropin not only enhances mitochondrial function via the activation of AMPK, leading to fatty acid oxidation and the inhibition of lipogenesis, but also regulates arterial NO via the activation of VEGF receptor 2 (VEGFR2) [41].

Multimerin 2 (MMR-2) is a pan-endothelial extracellular matrix protein that has been shown to interact in a highly specific manner with CD93 in the processes of angiogenesis and cell adhesion [42] and is correlated with coronary collateral artery angiogenesis following myocardial infarction [43]. CD93 also interacts with VE-cadherin, regulating its phosphorylation and turnover at endothelial junctions via the Rho/Rho kinase-dependent pathway [44]. Moreover, the MMR-2 ligand CD93, which can also be found in soluble form (sCD93), could stimulate FAK, ERK1/2, Akt, and eNOS, demonstrating its role as an angiogenic factor [45].

## 4. Biomarkers

In the final section, we review some of the early biomarkers associated with coronary collateral artery development following myocardial infarction, based mostly on clinical evidence and further detailed based on experimental data. This is of great importance in developing a fast, non-invasive method of determining the level of CCC development following MI (Table 1).

### 4.1. Cytokines and Growth Factors

Clinical studies have been performed on patients with stable CAD and chronic total occlusions (CTOs) using plasma isolated from collateral-specific versus coronary-specific blood samples. The data revealed higher concentrations of MCP-1, TGF-β, bFGF, and MIF, while stem cell factor (SCF) and stem cell growth factor-β (SCGF-β) were significantly downregulated in the collateral samples [53].

In another clinical study in patients with a successful percutaneous coronary intervention (PCI) for a CTO, the expression of several growth factors was assessed. The measurement of bFGF, MCP-1, TGF-β, and PIGF resulted in a correlation between the continuous release of bFGF and the duration of occlusion and collateral function, while FSS was related to the release of MCP-1, TGF-β, and PIGF and the presence of diabetes [58].

A clinical study on 196 patients showed a significant correlation between type II diabetes mellitus, hypertension, and serum VEGF levels when compared with collateral scores obtained by angiographic analysis (Rentrop grading system), but no significant correlation with total blood cholesterol levels [46]. VEGF has also been shown to increase the endothelial progenitor cell (EPC) count for a short period after the administration of VEGF in 165 plasmid constructs in a gene therapy clinical trial [59].

Growth differentiation factor 15 (GDF-15), a member of the TGF-β superfamily of growth factors, has been reported to have prognostic predictive value in coronary artery disease in a study on 210 patients. A positive correlation was found between the Rentrop grade and GDF-15, suggesting that GDF-15 levels may increase with the extent of collateral formation [49].

A clinical study found that the levels of MCP-1 were significantly increased during the early phase of AMI in patients with well-developed CCC when compared with patients with absent CCC [60].

A correlation has also been found between the severity of coronary stenosis and occlusion and the expression of stromal cell-derived factor 1 (SDF-1/CXCL12), as well as IP-10 (CXCL10) [61], both acting as chemoattractants for lymphocytes, monocytes, and NK cells [62]; however, SDF-1 has been shown to have angiogenic effects, while IP-10 exhibited angiostatic effects [63].

In a clinical study, a correlation between angiopoietin 1 (Ang-1), angiopoietin 2 (Ang-2), and calprotectin has been reported. Low levels of Ang-1 and calprotectin and higher levels of Ang-2 were associated with well-developed CCC [47].

Moreover, a clinical study revealed that Adropin levels were positively correlated with good CCC development in patients with chronic coronary syndrome (CCS) [48].

A clinical study on 88 subacute STEMI patients with either a right or left total coronary occlusion revealed a significant correlation between high MMR-2 expression and good CCC development specifically for patients with right coronary occlusions. Moreover, the values for the specific MI markers troponin T and BNP were higher in the poor CCC development group [43].

Other important markers for a well-developed collateral flow that do not fall into the category of cytokines or growth factors are bilirubin and Fe, which have been correlated with the activity of HO-1, peaking at 18–21 h after an interventional therapy in a clinical study conducted on 41 AMI patients [64].

### 4.2. microRNAs

Several microRNAs (miRs) have been reported to have significantly elevated levels in patients with insufficient collateral network development and symptoms of angina pectoris for ≥4 weeks and a CTO in a coronary artery, such as miR-423-5p, miR-10b, miR-30d, and miR-126, which could act as biomarkers in CTO patients with low collateral capacity [54]. Although associated with angiogenesis in vitro [65], it has been shown that the overexpression or deficiency of miR-126 could impede vascular remodeling, which is necessary for collateral development [51]. Moreover, miR-126 expression was found to be significantly decreased in patients with AMI as early as 4 h post-onset of AMI [66]. However, before miRs can be used as efficient biomarkers, coexisting parameters and pathological complications should be taken into consideration, such as T2DM and leukocyte counts, which can affect angiogenic mechanisms [67]. miR-423-5p has been associated with the induction of apoptosis in cardiomyocytes [68] and ferroptosis [69]. Experimentally, it has been shown that miR-423-5p inhibits EphrinA3 (EFNA3), thus promoting angiogenesis [70]. miR-10b is involved in blocking hypoxia-induced cardiomyocyte apoptosis via phosphatase and tensin homolog (PTEN) and the hypoxia-inducible factor 1α (HIF-1α) pathway [71]. Moreover, experimental data have shown that miR-10b can also promote embryonic stem cell-derived cardiomyocyte proliferation via the LATS1 gene, which is a major component of the Hippo signaling pathway [72]. In the context of myocardial infarction, miR-30d is expressed in extracellular vesicles secreted by cardiomyocytes as paracrine signals in the cross-talk with fibroblasts, which regulates Atg16-blocking autophagic processes [73]. miR-126, under hypoxic conditions, is involved in the function of endothelial progenitor cells via the PIK3 regulation subunit 2 (PIK3R2)/VEGF axis, promoting angiogenesis [65,66,74]. miR-155 expression, associated with VCAM-1, was also found to be higher in patients with poor CCC 2 days after diagnostic angiography in a study performed on a group of 78 CAD patients (34 with good CCC versus 44 with poor CCC) with at least one major coronary occlusion or stenosis [55]. miR-155 is secreted and packaged in exosomes by macrophages, and it has been associated with suppressing fibroblast proliferation and promoting inflammation during cardiac injury [55,75,76].

Another clinical study on 26 patients with symptoms of angina pectoris for ≥4 weeks and a CTO in a coronary artery revealed lower expression of miR-339-5p in the stimulated monocytes of patients with poor collateral capacity versus good collateral capacity [77]. A clinical study on patients with heart failure with a reduced ejection fraction revealed that miR-339-5p may be involved in ventricular remodeling via NOD-like receptor family CARD domain containing 5 (NLRC5), a regulator of immune and inflammatory responses, and also interacts with the PI3K/Akt signaling pathway [78].

In a recent study, the plasma levels of miR-210 from 253 participants were positively associated with coronary collateral circulation development. miR-210 mediates the influences of VEGF-A and EphrinA3 on CCC formation through mechanisms that have yet to be fully determined [50]. One such possible mechanism could be through miR-210’s inhibitory effect on APT2A2. miR-210 is upregulated under chronic hypoxic conditions by HIFs and directly targets APT2A2, resulting in increased cytosolic Ca^2+^ [79]. Another mechanism could be through the inhibition of MXD1, a transcriptional repressor of MYC [80]. Moreover, miR-210 is involved in the inhibition of mitochondrial energy production and reduces mitochondrial ROS in the heart during I/R injury by targeting glycerol-3-phosphate dehydrogenase (GPD2) [81]. In vitro experiments revealed that miR-210 can be expressed as early as 4 h [82] and has a half-life of approximately 5 days [83].

miR-21 and miR-26a have also been positively associated with a high collateral flow index (CFI) in patients with well-developed CCC [51]. Although miR-21, miR-26a, and miR-146a are important diagnostic markers for myocardial infarction [84,85], in vivo studies showed that prolonged exposure to miR-21 had detrimental effects on CCC development in metabolic syndrome animal models following repetitive coronary artery ischemia by stimulating the abnormal cell proliferation of vSMCs and inhibiting neutrophil apoptosis [86,87], suggesting a time-dependent mechanism for miR-21. Moreover, an in vitro study showed increased expression at 6 and 24 h after AMI in the border areas of infarcted rat hearts, but decreased expression in infarct areas [88]. A study on 14 STEMI and 17 NSTEMI patients that underwent coronary angiography and PCI with chest pain onset of a less than 4 h duration revealed an increase in miR-26a-1 plasma levels following AMI (at 4 h after the onset of symptoms), which was correlated with miR-146a and miR-199a-1 levels [89]. Another study on 78 CAD patients (34 with good CCC and 44 with poor CCC) revealed a positive correlation between the higher expression of miR-146a and good CCC, while the lower expression of miR-146a was correlated with poor CCC [90]. miR-21 is involved in inhibiting apoptosis via PDCD4/AP-1 [91,92] and promoting angiogenesis via PTEN/Akt signaling and also stimulating the expression of VEGF [93]. miR-21-enriched extracellular vesicles have been shown to restore cardiac function after myocardial infarction [94] and prevent excessive inflammation and cardiac dysfunction [95]. In experimental conditions, miR-26a inhibits myocardial cell apoptosis after AMI by activating the GSK-3β signaling pathway [96], as well as targeting PTEN via the JAK/STAT pathway [97]. Moreover, in both experimental mice and in humans, miR-26a regulates angiogenesis by targeting endothelial cell bone morphogenic protein (BMP)/SMAD1 signaling [98]. A study based on data obtained from seven STEMI patients revealed that miR-146a has a role in the inflammatory response by targeting S100A12, a calcium-binding protein expressed primarily by neutrophils that acts a pro-inflammatory danger signal (DAMP) [99].

Another recent study on 68 patients (22 CAD and 46 CTOs), with samples collected within 24 h of hospitalization, revealed the downregulated expression of miR-329, miR-494, and miR-495 in the good CCC group compared with the poor CCC group [52]. Experimentally, it was shown that miR-329 suppresses angiogenesis by directly targeting the endothelial cell marker CD146, which facilitates a response to VEGF-induced SRC kinase family (SKF)/p38 mitogen-activated protein kinase (MAPK)/NF-kB activation [100]. miR-494 was shown to be involved in regulating the PI3K/Akt/mTOR signaling pathway via targeting SIRT1 in experimental in vitro conditions [101], while, in experimental in vivo conditions, it was demonstrated that it can target the PTEN, ROCK1, and CaMKIIδ pro-apoptotic proteins, as well as the FGFR2 and LIF anti-apoptotic proteins [102]. miR-495 has been shown to facilitate apoptosis in experimental conditions by targeting Tenascin-C (TNC) [103] and to mediate inflammation by targeting pyrin domain-containing 3 (NLRP3) [104]. Moreover, low expression levels of miR-663 and let-7d were associated with high CCC development in the blood samples of patients with CTOs [51]. In experimental conditions, it was revealed that miR-663 blocked oscillatory shear stress-induced monocyte adhesion, while, in high laminar shear stress, it increased monocyte adhesion [105]. Other experimental studies have shown that let-7d modulates the proliferation, migration, and tubulogenesis of endothelial cells by directly targeting interferon-induced protein 44 (IFI44L) [106]. miR-503, which presents anti-angiogenic effects via the inhibition of VEGF-A, was also negatively correlated with VEGF-A and the Rentrop grade, and thus good CCC, in a study conducted on 168 patients (good CCC/Rentrop grade 2 or 3, *n* = 76, versus poor CCC/Rentrop grade 0 or 1, *n* = 92) [56].

AMI patients have also shown elevated levels of miR-181a, which could act as a myocardial infarction marker, having significantly elevated levels in plasma at 6 h, 12 h, and 24 h after the onset of AMI symptoms, with increased levels in the circulation at 6 h post-AMI and peaking at 24 h post-AMI [107], and it has also been correlated with a protective role in animal models [108]. It has also been suggested that miR-181c levels may be predictive of severe macrovascular complications, impairing angiogenesis in diabetic patients via direct or indirect targets such as HIF1-α and VEGF-A [109]. Although they are important biomarkers in MI and CVDs, miR-181 members (miR-181a, miR-181b, miR-181c, miR-181d) may have different and even opposite roles in certain pathological conditions, with a multitude of targets [110].

miR-379 participates in activating the MAPK/JNK/p38 pathway by downregulating DUSP1 expression [111], which is involved in cell survival, proliferation, and migration. A recent study showed that the DUSP1-JNK1/2 pathway may be involved in TRIM11’s cardioprotective effect against I/R injury in AC16 cardiomyocytes in vitro [112]. In vivo experiments using DUSP1 transgenic mice showed that the downregulated DUSP1 expression following I/R injury allows for the activation of the JNK pathway, which is also involved in the upregulation of mitochondrial fission factor (Mff) and Bnip3 activation, which lead to mitochondrial fission/mitophagy [113]. Moreover, decreased expression of circulating miR-379 was found in patients’ plasma after AMI, suggesting a negative correlation with myocardial infarction [114].

In vivo studies in mice revealed that miR-143 expression is induced by TGF-β in SMCs via serum response factor (SRF) and myocardin, which directly affects Col5a2 by downregulating its expression during collateral growth [115].

## 5. Discussion

Although there is a plethora of molecules involved in the arteriogenic development of CCC, many are also involved in molecular events post-MI, especially in the early phases. This poses a challenge in identifying a clear set of biomarkers for the presence of well-developed CCC. Moreover, a clearer distinction between angiogenesis (capillary sprouting) and arteriogenesis (collateral maturation) needs to be established. For example, several biomarkers could be used to achieve this distinction (Table 2).

A panel composed of a multitude of biomarkers, both proteic and molecular, could give clear insight into the presence of the arteriogenic process following MI in patients with well-developed CCC. Such a panel could encompass a specific combination of both positive and negative biomarkers, and several markers for CCC development show promising potential, such as positive markers Adropin, sCD93 (as a potential ligand for MMR-2), GDF-15 (a member of the TGF-β superfamily), Ang-2, and high expression of miR-210, combined with low expression of miR-329, miR-494, and miR-495, along with negative markers such as Ang-1, calprotectin, and high expression of miR-10b, miR-30d, miR-126, and miR-423-5p. Moreover, the importance of the Nrf2/HO-1 antioxidant system cannot be overlooked. HO-1 can have both pro- and anti-inflammatory effects, so we hypothesize that it might be involved in both the initiation of arteriogenesis, due to its pro-inflammatory action, and the cessation of inflammation once the arteries have been developed, based on its anti-inflammatory effect. This would make HO-1 a promising biomarker for arteriogenesis; however, other pathological complications might influence HO-1 expression, thus posing a challenge in using it as a single biomarker. It would also be beneficial to investigate the correlation of miR-143 and miR-379 expression in relation to CCC development based on their roles in arteriogenesis.

Several microRNAs have been identified as myocardial infarction biomarkers, with a detection time of between 6 and 24 h, such as miR-30a and miR-195, with a peak at 8 h and a decrease in expression after 12 h [116], as well as miR-122-5p with a peak at 8 h [117] and miR-181a with increased expression at 6 h and a peak at 24 h [107]. miR-21 has also been proposed as a potential early AMI biomarker, with a detection time as early as 6 to 72 h [118]. However, these biomarkers have only been correlated with AMI and not CCC development. As such, these markers already possess clinical value as potential early MI diagnostic markers, but they offer no insight into the CCC development level. miR-155, however, has been correlated with CCC development and has a reported detection time of 48 h [55] (Table 3). Therefore, adding the insight provided by early arteriogenesis markers into CCC development to the diagnostic value of early MI markers would enhance the diagnostic precision.

microRNAs, carried inside extracellular vesicles such as microparticles and exosomes as paracrine signals, can offer insight into the cellular and molecular processes taking place during the arteriogenesis of the collateral coronary arteries after myocardial infarction. Moreover, miRs such as miR-21, miR-126, and the miR-181 family have been shown to be involved in regulating endothelial junction proteins [119].

## 6. Conclusions

In conclusion, it is important from a clinical perspective to have a well-established molecular phenotypic profile of microRNAs that are produced very early after myocardial infarction in order to quickly determine the level of collateral coronary artery development. Therefore, by focusing on the role of microRNAs in the arteriogenic process, we propose the use of a mixed biomarker panel composed of early myocardial infarction biomarkers and early markers involved in the arteriogenesis process, which could be distinguished from markers involved in general angiogenesis. This profile would be completed and validated in correlation with cytokines and growth factors associated with the pathology and CCC development. As an example, the panel could be composed of several early myocardial infarction markers, such as miR-21, miR-30a, miR-122-5p, and miR-181a, followed by markers associated with CCC, such as miR-26a, miR-210, miR-146a, and miR-155. Ultimately, we propose markers that could be directly correlated with the arteriogenesis process, such as miR-143 and miR-379. This panel would be associated with a myocardial infarction marker such as cardiac troponin T (cTnI), followed by a marker that is associated with well-developed CCC and/or arteriogenesis, such as MCP-1, TGF-β, Ang1, Adropin, or MMR-2. As such, we propose the use of a mixed panel composed of a selection of MI early markers for diagnostic purposes (such as miR-30a, miR-195, miR-122-5p, miR-181a, or miR-21) and a selection of arteriogenesis early markers for the evaluation of the CCC development level (such as miR-155). This hypothesis needs to be tested with studies that identify early markers correlated with CCC development, rather than CCC development markers expressed later in the complex arteriogenesis process. For this purpose, we propose that the investigated early markers (Table 4) should be correlated with other biochemical markers, such as cytokines and growth factors, that have already been associated with CCC development to obtain a stronger correlation and improved insight into the arteriogenesis process following MI. However, due to the dynamic process of arteriogenesis, there are still limitations in current studies that need to be addressed in the future, such as the time of expression for some of the early biomarkers potentially associated with CCC development.

Together, these data would provide a panel of early markers of clinical importance that could have a predictive role in regard to the CCC development level, which can be assessed shortly after a myocardial infarction in a non-invasive manner and with a relatively low cost.

As such, it is important to have a well-established panel of both positive and negative biomarkers correlated with arteriogenesis, as a primary mechanism of CCC development, that can be detected as early as possible and in a non-invasive manner so as to better design possible therapeutic strategies.

## Figures and Tables

**Table 1:** Markers for coronary collateral circulation development in pathological conditions.

Biomarker	Pathology	CCC Assessment Criteria	Expression	Sample Type	Lot Size	Reference
**Good CCC**						
Biochemical markers						
VEGF	ACS (no previous PCI)	Coronary angiography	Upregulated	Serum	196 patients (110 T2DM/86 non-T2DM)	[46]
Ang2	CAD	Coronary angiography	Upregulated	Serum	147 patients (79 poor CCC/68 good CCC)	[47]
Adropin	CCS	Coronary angiography	Upregulated	Serum	102 patients (total occlusion: 50 poor CCC/52 good CCC)	[48]
GDF-15	CAD (no previous PCI)	Coronary angiography	Upregulated	Plasma	201 patients (66 CCC present/81 CCC absent)	[49]
EphrinA3	CAD	Coronary angiography	Downregulated	Plasma	253 patients (99 poor CCC/154 good CCC)	[50]
Multimerin 2	Subacute STEMI/CTO	Coronary angiography	Upregulated	Serum	88 patients (44 poor CCC/44 good CCC)	[43]
Molecular markers						
miR-210	CAD	Coronary angiography	Upregulated	Plasma	253 patients (99 poor CCC/154 good CCC)	[50]
miR-21	CAD/CTO	Coronary angiography	Upregulated	Blood	63 patients (19 poor CCC/17 good CCC)	[51]
miR-26a	CAD/CTO	Coronary angiography	Upregulated	Blood	63 patients (19 poor CCC/17 good CCC)	[51]
miR-146a	CAD	Collateral connection grade	Upregulated	Plasma	68 patients (22 CAD/46 CTO)	[52]
miR-329	CAD/CTO	Collateral connection grade	Downregulated	Plasma	68 patients (22 CAD/46 CTO)	[52]
miR-494	CAD/CTO	Collateral connection grade	Downregulated	Plasma	68 patients (22 CAD/46 CTO)	[52]
miR-495	CAD/CTO	Collateral connection grade	Downregulated	Plasma	68 patients (22 CAD/46 CTO)	[52]
miR-633	CAD/CTO	Coronary angiography	Downregulated	Blood	63 patients (19 poor CCC/17 good CCC)	[51]
let-7d	CAD/CTO	Coronary angiography	Downregulated	Blood	63 patients (19 poor CCC/17 good CCC)	[51]
**Poor CCC**						
Biochemical markers						
MCP-1	CAD	Collateral flow index	Upregulated	Collateral artery plasma	60 patients (25 non-total occlusion/35 total occlusion)	[53]
TGF-β	CAD	Collateral flow index	Upregulated	Collateral artery plasma	60 patients (25 non-total occlusion/35 total occlusion)	[53]
bFGF	CAD	Collateral flow index	Upregulated	Collateral artery plasma	60 patients (25 non-total occlusion/35 total occlusion)	[53]
MIF	CAD	Collateral flow index	Upregulated	Collateral artery plasma	60 patients (25 non-total occlusion/35 total occlusion)	[53]
SCF	CAD	Collateral flow index	Downregulated	Collateral artery plasma	60 patients (25 non-total occlusion/35 total occlusion)	[53]
SCGF-β	CAD	Collateral flow index	Downregulated	Collateral artery plasma	60 patients (25 non-total occlusion/35 total occlusion)	[53]
Ang1	CAD	Coronary angiography	Upregulated	Serum	147 patients (79 poor CCC/68 good CCC)	[47]
Calprotectin	CAD	Coronary angiography	Upregulated	Serum	147 patients (79 poor CCC/68 good CCC)	[47]
Molecular markers						
miR-10b	CTO	Collateral flow index	Upregulated	Aortic plasma	41 patients (with poor CCC)	[54]
miR-30d	CTO	Collateral flow index	Upregulated	Aortic plasma	41 patients (with poor CCC)	[54]
miR-126	CTO	Collateral flow index	Upregulated	Aortic plasma	41 patients (with poor CCC)	[54]
miR-155	CAD	Coronary angiography	Upregulated	Plasma	78 patients (44 poor CCC/34 good CCC)	[55]
miR-423-5p	CTO	Collateral flow index	Upregulated	Aortic plasma	41 patients (with poor CCC)	[54]
miR-503	CAD	Coronary angiography	Upregulated	Blood	168 (92 poor CCC/76 good CCC)	[56]
mir-339-5p (in vitro)	PCI after CTO	Collateral flow index	Downregulated	Isolated monocytes from arterial blood, in vitro intracellular expression	26 patients (12 poor CCC/14 good CCC)	[57]

**Table 2:** Potential biomarkers that could distinguish angiogenesis from arteriogenesis.

Process	Biomarker Type	Biomarkers
Angiogenesis	Molecular	miR-26a
		miR-146a
		miR-155
		miR-210
	Serological	VEGF
Arteriogenesis	Molecular	miR-143
		miR-379
	Serological	TGF-β
		MCP-1

**Table 3:** Time of expression of MI and potential CCC development for early microRNA biomarkers.

Biomarker	Pathology	Detection Time	Lot Size	Reference
miR-26a	AMI	4 h	31 patients (14 STEMI/17 NSTEMI)	[89]
miR-30a	AMI	6–24 h	18 patients	[116]
miR-122-5p	AMI	4–24 h	50 patients	[117]
miR-126	AMI	4 h	17 patients	[66]
miR-146a	AMI	4 h	31 patients (14 STEMI/17 NSTEMI)	[89]
miR-155	CAD	48 h	78 patients (44 poor CCC/34 good CCC)	[55]
miR-181	AMI/UA	6–24 h	120 patients (60 AMI/60 UA)	[107]
miR-195	AMI	6–24 h	18 patients	[116]
miR-329	CAD/CTO	24 h	68 patients (22 CAD/46 CTO)	[52]
miR-494	CAD/CTO	24 h	68 patients (22 CAD/46 CTO)	[52]
miR-495	CAD/CTO	24 h	68 patients (22 CAD/46 CTO)	[52]

**Table 4:** Proposed molecular biomarker panel for potential CCC development evaluation.

	**Good CCC**				**Poor CCC**			
Time of Expression	4 h	24 h	48 h	N/A	4 h	24 h	48 h	N/A
Positive	miR-26a			miR-210	miR-126			miR-10b
	miR-146a						miR-155	miR-503
Negative		miR-329						miR-399-5p
		miR-494						
		miR-495						

## Data Availability

No new data were created or analyzed in this study. Data sharing is not applicable to this article.

## Abbreviations

The following abbreviations are used in this manuscript:

CVD	Cardiovascular disease
MI	Myocardial infarction
CAD	Coronary artery disease
AMI	Acute myocardial infarction
ASC	Acute coronary syndrome
CCC	Coronary collateral circulation
FSS	Fluid shear stress
ECs	Endothelial cells
SMCs	Smooth muscle cells
MCP-1	Monocyte chemoattractant protein-1
ICAM-1	Intracellular adhesion molecule-1
VCAM-1	Vascular cell adhesion molecule-1
VEGF	Vascular endothelial growth factor
bFGF	Basic fibroblast growth factor
PDGF	Platelet-derived growth factor
NO	Nitric oxide
eNOS	Nitric oxide synthase
Egr-1	Early growth response 1
TGF-β	Transforming growth factor beta
MMPs	Metalloproteinases
ECP	External counterpulsation therapy
PMPs	Platelet-derived microparticles
AMPK	AMP-activated protein kinase
TRPV4	Transient receptor potential vanilloid
AP-1	Activated protein 1
DUSP1	Dual-specificity protein phosphatase 1
PLGF	Placental growth factor
MPs	Microparticles
AmotL2	Angiomotin-like 2
TAK1	TGF-β-activated kinase 1
α-TAT1	α-tubulin acetyltransferase 1
GSK-3β	Glycogen synthase kinase-3β
FAK	Integrin–focal adhesion kinase
JNK	c-Jun N-terminal kinase
VSMCs	Vascular smooth muscle cells
ECM	Extracellular matrix
MMR-2	Multimerin 2
CTOs	Chronic total occlusions
SCF	Stem cell factor
SCGF-β	Stem cell growth factor-β
PCI	Percutaneous coronary intervention
EPCs	Endothelial progenitor cells
GDF-15	Growth differentiation factor 15
SDF-1	Stromal cell-derived factor 1
Ang-1	Angiopoietin 1
Ang-2	Angiopoietin 2
CCS	Chronic coronary syndrome
miRs	microRNAs
PTEN	Phosphatase and tensin homolog
HIF-1α	Hypoxia-inducible factor 1α
PIK3R2	PIK3 regulation subunit 2
GPD2	Glycerol-3-phosphate dehydrogenase
CFI	Collateral flow index
BMP	Bone morphogenic protein
DAMP	Danger-associated molecular pattern
SKF	SRC kinase family
MAPK	Mitogen-activated protein kinase
TNC	Tenascin-C
NLRP3	Pyrin domain-containing 3
IFI44L	Interferon-induced protein 44
Mff	Mitochondrial fission factor
